# Análisis del perfil hematológico de los pacientes con la enfermedad por coronavirus 2019 (COVID-19)

**DOI:** 10.1515/almed-2022-0102

**Published:** 2022-12-21

**Authors:** Felisia Setio, Darwati Muhadi, Asvin Nurulita, Mansyur Arif, Irawaty Djaharuddin, Arifin Seweng

**Affiliations:** Hasanuddin University, Makassar, Indonesia; Dr. Wahidin Sudirohusodo Hospital, Makassar, Indonesia

**Keywords:** comorbilidad; COVID-19, inflamación, nivel de Ct, perfil hematológico, severidad

## Abstract

**Objetivos:**

Se han propuesto varios parámetros hematológicos como marcadores de gravedad de la COVID-19. Aún no se han realizado estudios en Makassar (Indonesia), para evaluar potenciales diferencias en el perfil hematológico de los pacientes según la gravedad de la enfermedad y las comorbilidades que presentan. Así mismo, tampoco se ha estudiado la correlación entre perfil hematológico y umbral de número de ciclos (Ct). El objetivo de este estudio es investigar posibles diferencias en el perfil hematológico de los pacientes con COVID-19 según la gravedad de la enfermedad y sus comorbilidades, así como determinar la posible correlación entre el perfil hematológico y el Ct en estos pacientes.

**Métodos:**

Se realizó un estudio retrospectivo transversal de pacientes con COVID-19 hospitalizados en el hospital del Dr. Wahidin Sudirohusodo en Makassar entre junio y agosto de 2020. Los datos sobre el perfil hematológico, niveles de Ct, comorbilidades y gravedad de la COVID-19 se extrajeron de la base de datos del hospital.

**Resultados:**

De los 217 pacientes, 102 (47%) eran hombres, frente a 115 (53%) mujeres. El número de pacientes con enfermedad leve o moderada fue de 127 (58.5%) mientras que 90 pacientes presentaban enfermedad grave (41.5%). En total, 143 pacientes (65%) no tenían comorbilidades, mientras que 74 pacientes (35%) sí las tenían. La amplitud de la distribución eritrocitaria, el recuento leucocitario, neutrófilos y monocitos, y la relación neutrófilos-linfocitos fueron significativamente superiores en los pacientes con enfermedad grave que en los que presentaban enfermedad leve o moderada (p<0,05). Así mismo, los pacientes graves presentaron un recuento de glóbulos rojos, hemoglobina, hematocrito, linfocitos y trombocitos significativamente inferior al de los pacientes con enfermedad leve o moderada. No se observaron diferencias significativas en el perfil hematológico según las comorbilidades, ni correlación alguna entre este y los niveles de Ct, excepto para el recuento de eosinófilos (r=0,161; p=0,018).

**Conclusiones:**

Nuestra hipótesis era que el perfil hematológico podría predecir la gravedad de la enfermedad en pacientes con COVID-19. Además, el recuento de eosinófilos debería tenerse en cuenta a la hora de predecir la infectividad de un paciente con COVID-19.

## Introducción

Según los datos de la Organización Mundial de la Salud (OMS), en la pandemia de enfermedad por coronavirus 2019 (COVID-19), causada por el Coronavirus 2 del Síndrome Respiratorio Agudo Severo (SARS-CoV-2) y que se inició en Wuhan (China), a fecha del 11 de octubre de 2020, se habían notificado 36.754.395 casos confirmados y 1.064.838 fallecimientos en todo el mundo, incluida Indonesia [1]. A esa misma fecha, el número de casos confirmados de COVID-19 en Indonesia era de 328,952, con 11,765 fallecimientos (3,6%). El distrito de Célebes Meridional (*Sulawesi,* en indonesio) es el quinto distrito con mayor número de casos de COVID-19, precedido por Yakarta (capital del país), Java Oriental, Java Occidental, y Java Medio, con un total de 16,399 casos confirmados y 431 fallecimientos (2.63%) [2].

El SARS-CoV-2 penetra en las células humanas mediante la unión de su proteína espícula con el receptor ACE2, que se expresa en múltiples órganos, como el pulmón, el riñón, y el endotelio intestinal y vascular. Las manifestaciones clínicas y la gravedad de la COVID-19 pueden variar sustancialmente. La mayoría de los pacientes con COVID-19 permanecen asintomáticos, o presentan síntomas de leve a moderados similares a los de una gripe o neumonía leve [3], [4], [5]. Sin embargo, alrededor del 14% de los pacientes desarrollan enfermedad grave y el 5% enfermedad crítica, con fallo respiratorio, septicemia, o disfunción multiorgánica. Los pacientes con comorbilidades como hipertensión, diabetes mellitus (DM), enfermedad cardiovascular, enfermedad renal crónica, o enfermedad respiratoria crónica, presentan un mayor riesgo de desarrollar COVID-19 grave [3, 6, 7].

Entre las pruebas analíticas normalmente empleadas en nuestro distrito para evaluar a los pacientes con COVID-19, se encuentran el hemograma completo y el análisis molecular. Se han realizado estudios para analizar los perfiles hematológicos de pacientes con COVID-19, habiéndose identificado algunos parámetros con supuesto valor clínico en la COVID-19. Los pacientes con COVID-19 tienden a presentar leucocitosis, neutrofilia, linfopenia, eosinopenia y trombocitopenia. Se han documentado niveles de hemoglobina (Hb) en el límite inferior de normalidad en pacientes con COVID-19 grave, frente a los pacientes con enfermedad leve a moderada, [8–10]. Por otro lado, se ha aportado evidencia de que los pacientes con COVID-19 presentan una relación neutrófilo-linfocito (RNL) más alta que los pacientes sin COVID-19 [4]. Además, este parámetro (RNL) también podría tener valor pronóstico en pacientes con COVID-19 [4, 11]. De este modo, se ha documentado que los pacientes con COVID-19 con una RNL alta podrían presentar valores elevados de marcadores de inflamación como la interleucina-6 y el factor de necrosis tumoral alfa (TNF-α). Además, la amplitud de la distribución eritrocitaria (ADE), un marcador de anisocitosis en el hemograma, se ha asociado a un mayor riesgo de rehospitalización en pacientes con COVID-19 [12]. Así mismo, los pacientes con COVID-19 y comorbilidades como la DM y la hipertensión parecen presentar marcadores de inflamación significativamente superiores a los de los pacientes infectados sin comorbilidades [13, 14]. También se han señalado algunos perfiles hematológicos como factores predictivos de vulnerabilidad en pacientes diabéticos con COVID-19 [15]. De este modo, se puede afirmar que existe una relación entre los marcadores de inflamación en sangre y la COVID-19.

Otra de las pruebas analíticas empleadas es la reacción en cadena de la polimerasa con retrotrancripción (RT-PCR). El umbral de ciclo (Ct) se obtuvo a partir de la RT-PCR y mostró una relación inversa con la carga viral. La correlación entre el Ct y la gravedad de la enfermedad continúa siendo objeto de debate, ya que aún no se ha investigado suficientemente [16–19]. Tampoco se han analizado posibles diferencias en el Ct entre pacientes con COVID-19 con comorbilidades y los pacientes que no las tienen.

En el presente estudio, tratamos de analizar el valor clínico de estas dos pruebas analíticas ordinarias y describir el perfil hematológico de los pacientes con COVID-19, según la gravedad y comorbilidades del paciente. Así mismo, analizamos la posible relación entre el perfil hematológico y el Ct en los casos confirmados de COVID-19.

## Materiales y métodos

Se realizó un estudio retrospectivo y transversal de pacientes con COVID-19, confirmado mediante RT-PCR, mayores de 18 años, que fueron ingresados en el hospital Dr. Wahidin Sudirohusodo, en el distrito de Célebes Meridional (Sulawesi, Indonesia) entre junio y agosto de 2020. Los datos se extrajeron de la base de datos del hospital. En primer lugar, se anonimizó la información eliminando los datos personales (nombre y número de identificación). A continuación, de la historia clínica, se extrajeron los datos demográficos (edad, sexo y comorbilidades), los resultados del hemograma completo y el Ct en la RT-PCR obtenidos en el primer ingreso en urgencias de los pacientes con COVID-19. Se excluyó del estudio a los pacientes oncológicos, así como a aquellos cuya historia clínica estuviera incompleta.

El perfil hematológico, basado en los resultados del hemograma completo, incluía: hemoglobina, hematocrito, recuento de eritrocitos, amplitud de la distribución eritrocitaria (ADE), recuento de leucocitos, recuento absoluto de eosinófilos, recuento absoluto de basófilos, recuento absoluto de neutrófilos, recuento absoluto de linfocitos, recuento absoluto de monocitos y trombocitos, que se obtuvieron con el analizador de hematología automatizado Sysmex XN-1000 (Sysmex Corporation, Kobe, Japón). La RNL se obtuvo dividiendo el recuento absoluto de neutrófilos por el recuento absoluto de linfocitos. Según la gravedad de la COVID-19, se dividió a los pacientes en dos grupos: pacientes con enfermedad de leve a moderada y pacientes con enfermedad grave. La gravedad de la COVID-19 la determinaba el neumólogo de urgencias, siguiendo las Directrices para la prevención y el control de la COVID-19 de Indonesia (5^a^ revisión). A continuación, se determinaba la planta de hospitalización a la que se derivaba al paciente, bien la planta de enfermedades infecciosas, o la planta de cuidados intensivos para pacientes con enfermedades infecciosas. Los pacientes con COVID-19 de leve a moderada sin signos ni síntomas de neumonía grave fueron ingresados en la planta general de enfermedades infecciosas, mientras que los pacientes con COVID-19 grave y neumonía grave (dificultad respiratoria >30 respiraciones/minuto o saturación de oxígeno <90%) fueron derivados a la planta de cuidados intensivos para pacientes con enfermedades infecciosas. La presencia de COVID-19 se confirmó mediante la detección de ARN en la zona de los genes ORF1ab y/o N del SARS-CoV-2 (Sansure Biotech Inc, Hunan, China) en muestras de frotis nasofaríngeo mediante RT-PCR (Lightcycler^®^ 480 II, Roche Diagnostics, Mannheim, Alemania). Los análisis se realizaron en el Laboratorio de Patología Clínica del hospital Wahidin Sudirohusodo en la ciudad de Makassar (Indonesia). Los pacientes asintomáticos con diagnóstico confirmado de COVID-19 no fueron hospitalizados, por lo que no se les incluyó en el estudio. Por otro lado, se dividió a los pacientes en otros dos grupos, según la presencia o no de comorbilidades, esto es: COVID-19 sin comorbilidades, en caso de ausencia de comorbilidades, y COVID-19 con comorbilidades, en caso de presencia de una o más comorbilidades.

Los datos fueron analizados con el programa IBM SPSS Statistics (version 22.0, IBM Corp., Armonk, NY, EE.UU). El análisis de normalidad se realizó mediante la prueba de Kolmogorov-Smirnov. Se utilizó la prueba t para muestras independientes, para analizar posibles diferencias en el valor medio de los parámetros hematológicos analizados y el Ct, según el nivel de gravedad de la enfermedad y la presencia o no de comorbilidades. Por otro lado, se empleó la prueba de correlación de Pearson para analizar la correlación entre los parámetros hematológicos y el Ct en los casos confirmados de COVID-19. Un valor p<0,05 se consideró estadísticamente significativo. Este estudio fue aprobado por el Comité de Ética de la Facultad de Medicina de la Universidad de Hasanuddin, del hospital Dr. Wahidin Sudirohusodo, el Hospital Universitario Wahidin Sudirohusodo, y el Hospital Universitario Makassar y Hasanuddin de Makassar, con el código 518/UN4.6.4.5.31/PP36/2020.

## Resultados y discusión

En total, se incluyó a 221 pacientes mayores de 18 años con diagnóstico confirmado de COVID-19, ingresados en el hospital Dr. Wahidin Sudirohusodo entre junio de 2020 y agosto de 2020, que cumplieran los criterios de inclusión. Posteriormente, se excluyó a 4 pacientes, ya que eran pacientes oncológicos, por lo que finalmente se incluyó a 217 pacientes. En la Tabla 1 se muestran las características demográficas de los pacientes de este estudio.

**Tabla 1:** Características demográficas de los pacientes con COVID-19.

Criterios	Número (%)	
**Sexo**		
Hombre	102 (47%)	
Mujer	115 (53%)	

**Edad** (18–85 años)		
Media (SD) = 41.8 (15.3)		
>18–30 años	73 (34%)	
31–45 años	59 (27%)	
46–59 años	51 (24%)	
≥60 años	34 (15%)	

Leve-moderada	127 (58,5%)	
Grave	90 (41,5%)	
Sin comorbilidades	143 (65%)	
Con comorbilidades	74 (35%)	
Hipertensión	40 (18%)	
Diabetes mellitus	27 (12%)	
Enfermedad cardiovascular	15 (7%)	
Enfermedad renal crónica	13 (6%)	
Enfermedad	3 (1%)	
Cerebrovascular		
VIH-SIDA	3 (1%)	
Otros	11 (5%)	

	**Mínimo–máximo**	**Media (SD)**

Leucocitos, células/µL	2.700–32.400	9.563,13 (4.405,96)
Eritrocitos, ×10^6^ células/µL	2,00–6,63	4,47 (0,86)
Hb, g/dL	5,8–19,1	1,73 (2,53)
HCT, %	17–56	37,50 (7,20)
PLT, ×10^3^ sel/µL	48–651	307,59 (108,98)
ADE, %	11,4–24,0	13,77 (1,97)
Neutrófilos, células/µL	1.504–29.808	6.645,29 (4.399,95)
Linfocitos, células/µL	280–5.947	2.007,9 (950,55)
Monocitos, células/µL	143–5.798	684,15 (446,69)
Eosinófilos, células/µL	0–1.184	178,57 (176,39)
Basófilos, células/µL	0–202	40,36 (29,95)
RNL	0,72–41,87	5,08 (6,54)
Valores Ct	15,92–39,37	30,05 (5,39)

En la Tabla 2 se observan las diferencias en los perfiles hematológicos de los pacientes con enfermedad leve a moderada, frente a aquellos con enfermedad grave, así como entre pacientes con y sin comorbilidades. Los pacientes con COVID-19 grave mostraron valores superiores de leucocitos, ADE, neutrófilos, monocitos y RNL, frente al grupo de pacientes con enfermedad leve a moderada (p<0,05). Por otro lado, los niveles de eritrocitos, hemoglobina, hematocrito, plaquetas y linfocitos fueron significativamente inferiores en el grupo con enfermedad grave (p<0.05). No se observaron diferencias significativas en el recuento de eosinófilos y basófilos entre los dos grupos, según la gravedad de la enfermedad. Así mismo, no hubo diferencias significativas en el perfil hematológico de los pacientes, según la presencia o ausencia de comorbilidades.

**Tabla 2:** Diferencias en los perfiles hematológicos de los pacientes COVID-19 según su gravedad y la presencia de comorbilidades.

Parámetro perfiles hematológicos ordinarios	Gravedad del paciente			Presencia de comorbilidades		
	Leve a moderado (n=127)	Grave (n=90)	p-Value	Sin comorbilidades (n=143)	Con comorbilidades (n=74)	p-Valor
	Media (SD)	Media (SD)		Media (SD)	Media (SD)	
Leucocitos, células/µL	8.148,82 (2.712,78)	1.1558,89 (5.460,11)	<0,001	9.518,88 (4.618,72)	9.648,65 (3.991,54)	0,830
Eritrocitos, ×10^6^células/µL	4,83 (0,63)	3,96 (0,87)	<0,001	4,51 (0,84)	4,39 (0,89)	0,319
Hb, g/dL	13,67 (2,0)	11,39 (2,60)	<0,001	12,83 (2,52)	12,52 (2,54)	0,390
HCT, %	40,35 (5,32)	33,48 (7,60)	<0,001	37,78 (7,09)	36,97 (7,43)	0,444
PLT, ×10^3^ células/µL	323,10 (100,10)	285,71 (117,52)	0,012	308,70 (100,60)	305,46 (124,29)	0,847
ADE, %	13,36 (1,87)	14,36 (1,97)	<0,001	13,73 (1,97)	13,85 (1,98)	0,658
Neutrófilos, células/µL	4.883,54 (2.421,12)	9.131,51 (5.292,09)	<0,001	6.559,48 (4.649,82)	6.811,35 (3.896,57)	0,674
Linfocitos, células/µL	2.415,43 (863,51)	1.433,20 (752,45)	<0,001	2.084,55 (970,92)	1.860,23 (897,81)	0,092
Monocitos, células/µL	616,08 (227,18)	780,29 (628,70)	<0,001	648,13 (282,76)	753,86 (653,79)	0,187
Eosinófilos, células/µL	189,27 (135,78)	163,48 (221,40)	0,290	182,58 (181,72)	170,82 (166,54)	0,634
Basófilos, células/µL	42,93 (30,01)	36,94 (29,61)	0,147	41,99 (31,36)	37,47 (26,89)	0,270
RNL	2,36 (2,05)	8,93 (8,51)	<0,001	4,84 (6,56)	5,55 (6,53)	0,455

Prueba t para muestras independientes, significativo si p<0,05.

Tampoco se observó una correlación significativa entre los niveles de leucocitos, Hb, plaquetas, ADE, RNL, neutrófilos, linfocitos, monocitos y basófilos y los valores de Ct. Se observó una débil correlación positiva entre los niveles de eosinófilos y el Ct (r=0,161, p=0,018) (Tabla 3).

**Tabla 3:** Correlación entre los perfiles hematológicos y el Ct en los pacientes con COVID-19.

Parámetro perfiles hematológicos ordinarios	Ct	
	R	p-Valor
Leucocitos	0,036	0,599
Hb	−0,099	0,150
PLT	0,074	0,280
ADE	−0,041	0,549
NLR	0,016	0,810
Neutrófilos	0,000	0,999
Linfocitos	0,102	0,137
Monocitos	0,060	0,384
Eosinófilos	0,161	0,018
Basófilos	0,083	0,223

Coeficiente de correlación de Pearson, significativo cuando p<0,05.

No se hallaron diferencias estadísticamente significativas en el Ct entre los pacientes con COVID-19 leve a moderada y los pacientes con enfermedad grave ​​(p=0,526) ni entre pacientes COVID-19 con comorbilidades y aquellos que no las presentaban (p=0,831) (Tabla 4).

**Tabla 4:** Diferencias en el valor de Ct basado en la gravedad del paciente y la presencia de comorbilidades.

	Gravedad del paciente			Presencia de comorbilidades		
	Leve-moderado (n=127)	Grave (n=90)	p-Value	Sin comorbilidades (n=143)	Con comorbilidades (n=74)	p-Valor
	Media (SD)	Media (SD)		Media (SD)	Media (SD)	
Ct	29,86 (5,34)	30,33 (5,48)	0,526	29,99 (5,49)	30,16 (5,24)	0,831

Prueba t para muestras independientes, significativo cuando p<0,05.

Según la OMS y las Directrices para la prevención y el control de la COVID-19 de Indonesia (5^a^-Revisión), el 20% de pacientes desarrollaron enfermedad crítica, mientras que el 80% presentaron enfermedad leve a moderada. Según la gravedad de la COVID-19, observamos diferencias en las características de los pacientes. Así el 41,5% de los pacientes desarrollaron enfermedad grave, mientras que el 58.5% presentaban enfermedad leve a moderada. El elevado porcentaje de pacientes con COVID-19 grave se debe a que el hospital Dr Wahidin Sudirohusodo es el centro de referencia para pacientes COVID-19 del distrito de Célebes Meridional en Indonesia. La edad media de los pacientes con diagnóstico confirmado de COVID-19 fue de 41,8 ± 15,3 años, que es el grupo de edad de población activa. El 65% de los pacientes COVID-19 que fueron hospitalizados en el Hospital Dr. Wahidin Sudirohusodo no presentaban comorbilidades, frente al 35% que presentaban comorbilidades, siendo las más frecuentes la hipertensión (18%), la diabetes mellitus (12%), y las enfermedades cardiovasculares (7%) [2, 3, 20].

El valor medio de leucocitos, neutrófilos, y monocitos en los pacientes con COVID-19 grave fue superior al del grupo con enfermedad leve a moderada, mientras que los pacientes graves presentaron niveles de linfocitos significativamente inferiores. El elevado recuento leucocitario en pacientes graves podría deberse a coinfecciones bacterianas o a una respuesta inmune más acusada. Estos resultados coinciden con los del estudio realizado por Karthabil et al. [9], que observaron que el recuento de leucocitos suele ser normal en los pacientes con COVID-19, excepto en los pacientes con COVID-19 grave, que presentaban niveles superiores. Así mismo, los pacientes con COVID-19 grave mostraban niveles inferiores de linfocitos, e incluso linfopenia (recuento medio de linfocitos en pacientes graves, 1.433.20 células/µL), lo que coincide con los resultados de otros estudios (Karthabil et al. [9], Liu et al. [8], Guan et al. [6], Qin et al. [21], Yang et al. [22]). Este fenómeno lo explicaron Yang et al. [22] y Qin C et al. [21], que documentaron en los pacientes con COVID-19 grave una disminución en los niveles de linfocitos T reguladores y en el porcentaje de células T de memoria, así como porcentajes elevados de linfocitos T *naive*. Estos hallazgos indican una disfunción en la respuesta inflamatoria, que era más acusada en los pacientes graves, lo que aumentaba la gravedad del fallo orgánico. El estudio llevado a cabo por Yang et al. [22] y por Gustine et al. [23] revelaba que el SARS-CoV-2 puede infectar directamente a los linfocitos y macrófagos. La autopsia de los pacientes con COVID-19 con linfopenia revelaba una elevada muerte celular de linfocitos y macrófagos en los ganglios linfáticos y esplénicos. Esto puede ser debido a que los macrófagos activados expresan citocinas inflamatorias, lo que induce la necrosis y apóptosis de los linfocitos. Yang et al. [22] explicaron que el elevado recuento de neutrófilos en los casos graves de COVID-19, se debe a que las alteraciones en los linfocitos facilitan el desarrollo de infecciones bacterianas, lo que provoca la activación y el reclutamiento de neutrófilos en el torrente sanguíneo. Karthabil et al. [9], y Liu et al. [8] identificaron el recuento de neutrófilos como un factor predictivo de gravedad de la enfermedad [6, 8, 9, 21–23].

La relación neutrófilo-linfocito (RNL) combina la neutrofilia y la linfopenia, por lo que predice con mayor precisión la inflamación sistémica. Dado que se trata de una relación, se ve afectada en menor medida por factores preanalíticos, y se puede obtener fácilmente mediante una analítica ordinaria. La RNL se asocia con varias enfermedades inflamatorias, como la tiroiditis, colitis ulcerosa, diabetes mellitus no controlada, enfermedad del intestino irritable y, más recientemente, la infección por COVID-19 [4, 24], [25], [26], [27]. Además, existe evidencia sólida de que los pacientes con COVID-19 presentan una RNL alta, con un valor umbral de ≥3,13 para la categoría con mayor riesgo, y de <3,13 para la categoría de pacientes con menor riesgo de desarrollar enfermedad grave. Estos resultados coinciden con los de nuestro estudio, donde se obtuvo una RNL media de 8,93 en los casos graves, frente a 2,36 en los casos leves o moderados. Se observó una relación entre una RNL elevada y la elevación de algunos marcadores inflamatorios en pacientes con COVID-19 grave, como la proteína C-reactiva (PCR), la interleukina-6, TNF-α, y la ferritina sérica [4, 6, 8, 9, 21–23, 28].

Los casos graves presentaron niveles superiores de monocitos que los casos leves o moderados. Esto contradice los resultados del estudio de Yang et al. [22] y Khartabil et al. [9], que reportaron niveles inferiores de monocitos en los casos graves, ya que la activación de monocitos puede inducir la liberación de citocinas inflamatorias, que agravan el daño en el tejido pulmonar y en otros tejidos. El recuento de eosinófilos y basófilos fue inferior en los pacientes con COVID-19 grave, aunque las diferencias no fueron significativas. Esto se debe a las bajas concentraciones de eosinófilos y basófilos normales en sangre, ya que los investigadores no distinguieron a los pacientes COVID-19 críticos con septicemia. Por otro lado, también se ha estudiado la eosinopenia como marcador de septicemia [6, 8, 9, 21–23, 29].

Los pacientes con COVID-19 grave mostraron niveles inferiores de hemoglobina y niveles superiores de ADE, lo que sugiere que un proceso inflamatorio más acusado interfiere con el proceso de eritropoyesis [6, 8]. En este estudio, el recuento de plaquetas se mantuvo dentro de límites normales, aunque con niveles significativamente bajos en los pacientes con COVID-19 grave [30–32].

En este estudio, la presencia de comorbilidades no influyó en el perfil hematológico de los pacientes con COVID-19. Estos resultados coinciden con los del estudio realizado por Manson, que observó que los pacientes con COVID-19 con respuesta hiperinflamatoria al ingreso presentaban menos comorbilidades que aquellos que no presentaban hiperinflamación. Esto contrasta con el estudio de Zhou et al. [33]. Chrsitensen et al. [34], que observaron que relacionaron un mayor número de comorbilidades estaba con un peor pronóstico. Esto se debe a que, en este estudio, se compararon los perfiles hematológicos según la presencia o ausencia de comorbilidades en el momento del ingreso, pero no se realizó un seguimiento de la evolución del perfil hematológico hasta el alta o el fallecimiento del paciente [33], [34], [35].

En este estudio, no se hallaron diferencias estadísticamente significativas en el Ct según el nivel de gravedad de la enfermedad. Este hallazgo contrasta con los resultados del estudio de Rao et al. [16], que observaron que la determinación del Ct es un método indirecto para calcular el número de copias del coronavirus, existiendo una relación inversa entre el Ct y el nivel de gravedad de la enfermedad. Esto sugiere que un Ct bajo no indica necesariamente un mal pronóstico. Este resultado coincide con el estudio de Argyropoulos [36], que halló títulos de carga viral inferiores en los pacientes hospitalizados con COVID-19 que en los pacientes ambulatorios, y también indicaban el tiempo transcurrido desde el inicio de la infección. Por otro lado, Singanayagam y Young et al. [18] no observaron ninguna relación entre el Ct y el nivel de gravedad de la enfermedad. No hubo diferencias significativas en el Ct de los pacientes según la presencia o ausencia de comorbilidades. Esto indica que la presencia de comorbilidades no influye en la infectividad de una persona [16, 19, 36].

También confirmaron que no existía relación entre el Ct y los parámetros hematológicos, a excepción del número de eosinófilos. Estos resultados refuerzan la hipótesis de que el desarrollo de COVID-19 grave está más relacionado con la enfermedad inflamatoria y la desregulación del sistema inmune que con el Ct en el momento del ingreso. No obstante, basándonos en los resultados de este estudio, el recuento de eosinófilos podría ser útil a la hora de calcular la carga viral junto con el valor de Ct, aun existiendo una relación débil entre estos marcadores y el desarrollo de COVID-19 grave. Así, cuanto menor sea el nivel de Ct, mayor será el nivel de infectividad. Sin embargo, son necesarios más estudios de cohortes en una muestra más amplia de pacientes. Li et al. [37] observaron que el recuento de eosinófilos era significativamente inferior durante el diagnóstico inicial y la recuperación que en los pacientes con enfermedad COVID-19 grave. Además, el recuento de eosinófilos puede mejorar cuando se reduce la carga viral [16, 17, 37].

Este estudio subraya la importancia de varios parámetros hematológicos a la hora de predecir la gravedad de los pacientes COVID-19, y facilitar un manejo temprano y optimización del tratamiento. El hemograma completo es una prueba sencilla, económica y común que está disponible en la mayoría de los centros médicos y con un elevado valor clínico. En el cribado de la COVID-19, niveles inferiores de eosinófilos podrían ser un factor predictivo de un Ct inferior, lo que indica un mayor nivel de infectividad del paciente. En los pacientes con diagnóstico confirmado de COVID-19, se puede emplear la RNL como parámetro alternativo a la hora de evaluar el estado inflamatorio.

Este estudio presenta algunas limitaciones. En primer lugar, no se realizaron evaluaciones seriadas de los perfiles hematológicos y del Ct durante el tiempo de ingreso, por lo que no se pudieron analizar los cambios en la respuesta del organismo, que se manifiestan en forma de cambios en los perfiles hematológicos y en la dinámica de los valores de Ct. Además, no se evaluó la influencia de las comorbilidades en los resultados clínicos. El efecto de las comorbilidades en este estudio se analizó en su conjunto, pero no se analizó el efecto individual de cada comorbilidad en el perfil hematológico de los pacientes. Las patologías como el fallo renal crónico o la infección por VIH pueden afectar al perfil hematológico de los pacientes con COVID-19.

En conclusión, el perfil hematológico podría predecir el desarrollo de COVID-19 grave. Además, el recuento de eosinófilos debería tenerse en cuenta a la hora de estimar la infectividad de un paciente con COVID-19. Son necesarios más estudios de cohortes para evaluar la dinámica de los perfiles hematológicos en los pacientes con COVID-19.

## References

[1] (2020). WHO coronavirus diseases (COVID-19) Dashboard.

[2] Map D (2020). Task force for the acceleration of handling COVID-19 in Indonesia.

[3] WHO (2020). Clinical management of COVID-19: interim guidance.

[4] Aktas G (2021). Hematological predictors of novel coronavirus infection. Rev Assoc Med Bras.

[5] Wu Z, McGoogan JM (2020). Characteristics of and important lessons from the coronavirus disease 2019 (COVID-19) outbreak in China summary of a report of 72314 cases from the Chinese center for disease control and prevention. JAMA.

[6] Guan W, Ni Z, Hu Y, Liang W, Ou C, He J (2020). Clinical characteristic of coronavirus disease 2019 in China. N Engl J Med.

[7] Guan WJ, Liang WH, Zhao Y, Liang H, Chen Z, Li Y (2020). Comorbidity and its impact on 1590 patients with COVID-19 in China: a nationwide analysis. Eur Respir J.

[8] Liu X, Zhang R, He G (2020). Hematological findings in coronavirus diseases 2019: indications of progression of disease. Ann Hematol.

[9] Khartabil TA, Russcher H, Ven AD, Rijke YB (2020). A summary of diagnostic and prognostic value of hemocytometry markers in COVID-19 patients. Crit Rev Clin Lab Sci.

[10] Liao D, Zhou F, Luo L, Xu M, Wang H, Xia J (2020). Haematological characteristic and risk factors in the classification and prognostic evaluation of COVID-19: a retrospective cohort study. Lancet Haematol.

[11] Khalid A, Jaffar MA, Khan T, Lail RA, Ali S, Aktas G (2021). Hematological and biochemical parameters as diagnostic and prognostic markers in SARS-CoV-2 infected patients of Pakistan: a retrospective comparative analysis. Hematology.

[12] Atak Tel BM, Kahveci G, Bilgin S, Kurtkulagi O, Duman T, Demirkol M (2022). Haemoglobin and red cell distribution width levels in internal medicine patients indicate recurrent hospital admission during COVID-19. Fam Med Prim Care Rev.

[13] Guo W, Li M, Dong Y, Zhou H, Zhang Z, Tian C (2020). Diabetes is a risk factor for progression and prognosis of COVID-19. Diabetes Metab Res Rev.

[14] Huang S, Wang J, Liu F, Liu J, Cao G, Yang C (2020). COVID-19 Patients with hypertension have more severe disease: a multicenter retrospective observational study. Hypertens Res.

[15] Atak Tel BM, Bilgin S, Kurkulagi O, Kahveci G, Duman T, Sagdic T (2021). Frailty in diabetic subjects during COVID-19 and its association with HbA1c, mean platelet volume and monocyte/lymphocyte ratio. Clin Diabetology.

[16] Rao SN, Manissero D, Steele VR, Pareja J (2020). A narative systematic review of the clinical utility of cycle treshold value in the context of COVID-19. Infect Dis Ther.

[17] Huang JT, Ran RR, Lv ZH, Feng L, Ran C, Tong Y (2020). Chronological changes of viral shedding in adult inpatients with COVID-19 in wuhan, China. Clin Infect Dis.

[18] Young BE, Ong SW, Ng LF, Anderson D, Chia W, Chia P (2021). Viral dynamics and immune correlates of coronavirus disease 2019 (COVID-19) severity. Clin Infect Dis.

[19] Singanayagam A, Patel M, Charlett A, Bernal JL, Saliba V, Ellis J (2021). Duration of infectiousness and correlation with RT-PCR cycle threshold values in case of COVID-19, england, january to may 2020. Euro Surveill.

[20] Sugihantono A, Burhan E, Susanto AD, Damayanti T, Wiyono WH, Samuedro E (2020). Guidelines for prevention and control of coronavirus disease (COVID-19) 5th revision.

[21] Qin C, Zhou L, Hu Z, Zhang S, Yang S, Tao Y (2020). Dysregulation of immune response in patients with coronavirus 2019 (COVID-19) in wuhan, China. Clin Infec Dis.

[22] Yang L, Liu S, Liu J, Zhang Z, Wan X, Huang B (2020). Review article COVID-19: immunopathogenesis and immunotherapeutics. Sig Transduct Target Ther.

[23] Gustine JN, Jones D (2020). Review: immunopathology of hyperinflamation in COVID-19. Am J Pathol.

[24] Aktas G, Sit M, Dikbas O, Erkol H, Altinordu R, Erkus E (2017). Elevated neutrophil-to-lymphocyte ratio in the diagnosis of hashimoto’s thyroiditis. Rev Assoc Med Bras.

[25] Posul E, Yilmaz B, Aktas G, Kurt M (2015). Does neutrophil-to-lymphocyte ratio predict active ulcerative colitis?. Wien Klin Wochenschr.

[26] Bilgin S, Aktas G, Kocak MZ, Atak M, Kurtkulagi O, Duman T (2019). Association between novel inflammatory markers derived from hemogram indices and metabolic parameters in type 2 diabetic men. The Aging Male.

[27] Aktas G, Basaran E, Duman TT, Atak BM, Kurtkulagi O, Bilgin S (2020). Irritable bowel syndrome is associated with novel inflammatory markers derived from hemogram parameters. Fam Med Prim Care Rev.

[28] Manasathreya AV, Devi R (2022). The relation between neutrophil-lymphocyte ratio and inflammatory markers in assessing the severity of COVID-19 disease. Eur J Clin Med.

[29] Savitskiy A, Rudnov V, Bagin V (2015). Eosinophenia as a marker of sepsis and mortality in critically ill patients. Crit Care.

[30] Guglu E, Kocayigit H, Okan HG, Erkorkmaz U, Yurumez Y, Yaylaci S (2020). Effect of COVID-19 on platelet count and its indices. Rev Assoc Med Bras.

[31] Gong J, Ou J, Qiu X, Jie Y, Chen Y, Yuan L (2020). A Tool to early predict severe 2019-novel coronavirus pneumonia (COVID-19): a multicenter study using the risk nomogram in wuhan and guangdong, China. Clin Infect Dis.

[32] Salamanna F, Maglio M, Landini MP, Fini M (2020). Platelet functions and activities as potential hematologic parameters related to coronavirus disease 2019 (COVID-19). Platelets.

[33] Zhou W, Qin X, Hu X, Lu Y, Pan J (2020). Prognosis models for severe and critical COVID-19 based on the charlson and elixhauser comorbidity indices. Int J Med Sci.

[34] Christensen DM, Strange JE (2020). Charlson. Comorbidity index score and risk of severe outcome and death in Danish COVID-19 patients. J Gen Intern Med.

[35] Manson JJ, Crooks C, Naja M, Ledlie A, Goulden B, Liddle T (2020). COVID-19-associated hyperinflammation and escalation of patient care: a retrospective longitudinal cohort study. Lancet Rheumatol.

[36] Argyropoulus KV, Serrano A, Hu J, Black M, Feng X, Shen G (2020). Association of initial viral load in severe acute respiratory syndrome coronavirus 2 (SARS-CoV-2) patients with outcome and symptoms. Am J Pathol.

[37] Li X, Yin D, Yang Y, Bi C, Wang Z, Ma G (2021). Eosinophil: a nonnegligible predictor in COVID-19 re-positive patients. Front Immunol.

